# Nine–month nutritional intervention improves restoration of menses in young female athletes and ballet dancers

**DOI:** 10.1186/s12970-014-0052-9

**Published:** 2014-10-31

**Authors:** Karolina Łagowska, Karina Kapczuk, Jan Jeszka

**Affiliations:** Department of Human Nutrition and Hygiene, Poznan University of Life Sciences, Poznan, Poland; Department of Perinatology and Gynecology, Poznan University of Medical Sciences, Poznan, Poland

**Keywords:** Body composition, Metabolism, Nutrition

## Abstract

**Background:**

We hypothesized that an intervention designed to increase the energy and nutrient intake could serve as an efficacious method to restore normal menstrual functions in athletes and ballet dancers.

**Methods:**

In this study, a 9-month nutritional intervention (NI) was conducted in 21 dancers and 31 athletes with menstrual disorders. Analyses of the body composition were performed, and the levels of LH, FSH, P, E2, TSH, T, PRL, SHBG, leptin, resting metabolic rate (RMR), energy and nutrient intake, total energy expenditure were estimated. The NI was based on an individual diet. The effects of the NI were controlled after 3, 6 and 9 months of use.

**Results:**

The NI resulted in a significant change of the energy and nutrient intake. After 9 months, a significant increase in the LH level among dancers was observed, while in female athletes this effect was seen after 3 months of the NI use. The 9-month NI resulted in the restoration of regular menses in 3 dancers and 7 athletes, respectively. Women with regular cycles had a higher percentage of the fat mass (FM).

**Conclusions:**

A non-pharmacological intervention in female athletes and ballet dancers with menstrual disorders can restore regular menstrual cycles, although restoration of menses may take more than 1 year. An increase in the body fat mass may be one of the most important predictors of restoration of menses.

## Background

It has been shown that energy and nutrient values of daily diets (DD) and energy availability have an important place in the etiology of menstrual disorders in female athletes. Due to the start of physical training at a young age and high daily energy expenditure combined with strict weight limits ballet dancers are also in a high-risk group of such disturbances. Furthermore, the results of studies performed by other authors, regarding ballet dancers and female athletes from different sports disciplines, confirm that the knowledge on the potential effects of untreated menstruation disorders is limited [1,2].

There are several reports regarding the outcomes of a non-pharmacological nutritional intervention in female athletes with menstrual disorders. Arends et al. [3] indicated that a weight gain or BMI increase may be important predictors of menses restoration. These results were based on the outcomes taken from a 5-year retrospective study of 373 female athletes treated with non-pharmacological therapies, including an increased dietary intake and/or decreased exercise expenditure. In a case report, Mallinson et al. [4] compared and contrasted the responses of two exercising women with amenorrhea of varying duration to a 12-month nutritional intervention including an increased energy intake; however, without changes in the total energy expenditure (TEE) and training volume. The restoration of menses occurred on day 23 and 74 of the intervention for women with short- and long-term amenorrhea, respectively; however, the onset of ovulation and regular cycles was associated not only with an energy intake increase, but also with a weight gain increase. Those authors suggested that further research in larger samples is necessary to determine the primary contributors of menses resumption in exercising women with amenorrhea. Our study could be an answer to this suggestion. We previously identified 31 female athletes with hypothalamic menstrual disorders, in whom treatment with a nutritional intervention included an individual diet and nutritional recommendations without any change in the volume and intensity of training. Three months of a nutritional intervention resulted in a significant increase of energy and nutrient intakes, increase of energy availability and energy balance. Also the LH level and the FSH to LH ratio elevations, but without changes in the menses rhythm, were observed [5]. Then, we decided to include ballet dancers to this study and continue the controlled nutritional intervention to assess the extent to which long-term improvement in the nutritional status would allow for menstruation recovery. To our knowledge, this is the first study in which ballet dancers with menstrual disorders were treated with a non-pharmacological nutritional intervention.

## Methods

### Subjects

In this study, 45 well-trained female athletes with menstrual disorders (18 rowers, 12 synchronized swimmers, 15 triathlonists) from different sports clubs in Poznan and 27 ballet dancers from the Secondary Ballet School in Poznan were enrolled. The inclusion criteria were as follows: menstrual disorders within the last 12 months, at least 3-year training period, training session frequency > 4/wk., no serious medical conditions, no use of hormonal contraception or other medications that might interfere with the hypothalamic-pituitary-gonadal axis, no clinical diagnosis of eating disorders, no history of primary ovarian failure, hyperprolactinemia, thyroid dysfunction or polycystic ovary syndrome and non-smoking. Finally, 31 female athletes and 21 dancers completed a nine-month NI period (1 female athlete with hypothyroidism was excluded from the analysis, 2 athletes were excluded because of sports injuries, 11 withdrew their consent and 6 discontinued dropped out ballet school). A written informed consent was obtained from all participants and their parents. The study was approved by the Poznan Medical Ethics Committee (No.: 334/09).

### Menstrual status

Each subject completed a questionnaire. In the first part of the questionnaire, questions were focused on menstruation: age at menarche, length of menses and history of amenorrhea. The second part of the questionnaire referred to sport activities: age at the beginning of training, training period, number of training sessions per week, hours of training per day and per week. Primary amenorrhea was diagnosed in females without the onset of menses by the age of 15, while secondary amenorrhea was diagnosed in subjects without menstruation for a 6-month period. Menstrual periods occurring with intervals of more than 35 days were defined as oligomenorrhea [6]. Similarly to Mallinson et al. [4], we described the recovery of menstrual function as the resumption of menses followed by at least 2 menses of less than 36 days in duration. Each subject underwent a gynaecological evaluation, including pelvic ultrasound and luteinizing hormone (LH), follicle-stimulating hormone (FSH), progesterone (P), estradiol (E2), prolactin (PRL), thyroid-stimulating hormone (TSH), testosterone (T), and sex-hormone-binding globulin (SHBG) levels assessment in order to exclude independent causes of amenorrhea or oligomenorrhea (such as pregnancy, primary ovarian failure, hyperprolactinaemia, thyroid dysfunction or polycystic ovary syndrome). The serum leptin level was also measured. In subjects with possible polycystic ovary syndrome, hyperandrogenism (elevated testosterone levels or LH to FSH ratio >3:1), thyroid dysfunction (normal range for TSH was established at 0.4–4.0 mIU/L), hyperprolactinaemia (PRL >25 ng/mL), other examinations to diagnose or exclude other causes of amenorrhea and oligomenorrhea were also performed [7,8].

### Blood sampling and biochemical analyses

In subjects with irregular cycles, blood samples were collected between day 2 and 5 of the menstrual cycle (in the early follicular phase), and in amenorrheic subjects the samples were taken in a random manner. Blood samples were drawn between 6.00 a.m. and 9.00 a.m., following overnight fasting and rest. Subjects were instructed to abstain from caffeine and alcohol during a 24-hour period prior to blood sampling, and to refrain from strenuous exercise on the day of sampling. The levels of LH, FSH, E2, P, PRL, TSH, T and SHBG were measured by immunochemical methods using Chemiluminescent Microparticle Immunoassay (CMIA) and Microparticle Chemiflex Flexible assay protocols, and diagnostic sets and an ARCHITECT automatic analyser. The serum leptin levels were estimated using the Human Leptin Elisa by LINCO Research. All hormones levels were determined in a duplicate.

### Body weight and body composition measurements

Height and body weight (BW) were measured using an anthropometer coupled with a WPT 200 OC verified medical scale (Rad Wag). During measurements, all participants were dressed minimalistically, and the obtained results were rounded to the nearest 0.5 kg and 0.5 cm, respectively. The analysis of the body fat mass (FM) and fat-free mass (FFM) was performed in the morning, following overnight fasting, in subjects lying in a supine position, using BODYSTAT 1500, as described by Heyward et al. [9].

### Resting metabolic rate

Resting metabolic rate (RMR) was assessed by using a portable indirect calorimeter for 25 minutes (Cosmed K4b2, Cosmed, Italy). A face mask (Hans Rudolph, Kansas City, MO) covering the mouth and nose of the participant was attached to a bidirectional digital turbine flow-meter and fastened to the participant using a mesh hairnet with Velcro straps. To guarantee an airtight seal, a disposable gel seal (Hans Rudolph) was positioned between the inside of the face mask and the skin. The Cosmed K4b2 system was calibrated prior to each individual test according to the manufacturer’s guidelines. Breath-by-breath O_2_ and CO_2_ gas exchange was measured and recorded in the portable unit’s computer system. On completion of each test, the stored data were transferred to the Cosmed K4b2 version 6 computer software running on a Windows-based laptop computer. The data were then averaged over 15 second intervals and transferred to Microsoft *Excel* for further analysis. The morning before the RMR measurements, the Cosmed K4b2 was calibrated with a calibration gas mixture (16% O_2_, 5% CO_2_). The test was carried out with the participant in a comfortable supine position, at an environmental temperature of 21–22°C. All measurements were done in the morning (between 6 and 9 a.m.) following a 12 hours fast and a minimum of 8 hours of rest. The results of the RMR measurement were compared with the RMR predicted by the Harris-Benedict equation [10] and the RMR(kcal)/FFM(kg) ratio was also calculated. In anorexic women, a reduced ratio of measured REE value to REE value calculated with the use of Harris–Benedict prediction equation amounting to 0.60–0.80 during periods of low BW and prior to refeeding has been reported [11,12]. In this study, a ratio <0.90 correlated with energy deficiency.

### Total energy expenditure and energy availability

Similarly to study by Reguła et al. [13] and ours previously study [5], each subject was wearing a heart rate monitor (HR) (Polar Sport Tester, RS 400, Finland), during a 3-day period, in order to estimate the total energy expenditure (TEE). The 24-hour energy balance (EB) was calculated as the difference between the mean of 24-hour energy intake, taken from seven consecutive days, and the mean of TEE calculated on the basis of three-day measurement. Energy availability (EA) was calculated by subtracting the exercise energy expenditure (EEE) from the total daily energy intake, and was adjusted for the FFM (kg) [6].

### Nutritional intervention

Under the supervision of dieticians, dietary records from 7 consecutive days were obtained. Subjects had a regular contact with a registered dietician, who instructed them how to record a nutrient intake. All meals (including recipes and weights of products), snacks, beverages and fluids were recorded in a diary form using a Photographic Album of Products and Dishes [14]. DD were analysed with regard to their energy and nutrient levels (fat, protein, carbohydrate, calcium, vitamin D) using the *Dietician* computer software package, based on Polish food composition tables [15]. Next, all participants were informed about nutritional mistakes in their current diets and about consequences of nutritional deficiencies. Subsequently, as a part of the nutritional intervention (NI), we prepared an individual diet for each subject. During first months of nutritional intervention, the baseline energy value was increased by 20-30% per month, until zero energy balance and energy availability >40 kcal/kg FFM/d were obtained. Thus, the energy value of recommended diets ranged from 2500 to 3500 kcal per day. The wide range of values was dependant on individual features, such as: anthropometric measurements, age and sport discipline or actual training period and associated training intensity. Female triathlonists had the highest energy requirements, while in dancers the energy requirements were the lowest. The recommended level of nutrients intake was determined in accordance with the recommendations of the ACSM Female Athlete Triad Position Stand [6] and Jarosz et al. [16]. Also, the percentage of nutrients was mainly dependant on sport discipline, however female triathlonists received a diet providing minimum 60% of energy from carbohydrates. The percentage of energy taken from fats was 25-30% with saturated fats intake less than 10%; while proteins covered 16-18% of energy requirements, and at least one half of protein was of animal origin. During diet composition, the individual food preferences of each competitor and dancer, daily schedule, training hours, aversions and food intolerances were taken into account. However, highly-processed food, sweets, savoury snacks and fast foods were excluded. Diets were composed of 5 or 6 meals, including 3 main meals (breakfast, lunch, dinner) with additionally pre- and post training snacks. Also, an adequate fluid intake, including isotonic drinks, with a volume depending on quantity, length and intensity of trainings was considered. During dietary counselling sessions, issues concerning special foods for athletes, sports beverages, supplements, shopping tips, low-fat and low-calorie food, food preparation, dining out, iron, calcium and vitamins content in foods were also elaborated. After every month of NI use, the monitoring of following parameters was performed. Repeated assessments of TEE (1 day), EA, energy and nutrient values of DD (3 days) were conducted (data no shown). Next, after 3, 6 and 9 months NI effect follow-up was performed, and subsequently TEE (3 day), EA, energy and nutrient values of subject’s daily diets (DD) (7 days), LH, FSH, E2 and P levels were reassessed. Those data were used for the purpose of statistical analysis.

### Statistical analysis

Means and standard deviations of quantitative variables were calculated. The normality of the distribution was checked. Comparisons between data obtained pre and post 3-, 6- and 9-month time point of the nutritional intervention were carried out with the use of repeated measures analysis of variance. Comparisons between dancers and female athletes groups were carried out using a *t*-test for independent variables. Statistical analyses were performed using *Statistica 8.0* software (StatSoft, 2008). *P*-values below 0.05 level were considered statistically significant.

## Results

The characteristics of subjects who completed the study are shown in Table 1. The duration of training period differed significantly between two study groups, being significantly longer for dancers. The daily training load was also significantly higher in ballet dancers; and they also obtained significantly lower, predicted and measured, RMR values. RMR/FFM ratios *100% also differed significantly between two above mentioned study groups. The age at menarche and T level, between dancers and female athletes, were also significantly different. The serum leptin level did not differ significantly between the groups, but both dancers and athletes had a lower value than the predicted laboratory range. In the group of ballet dancers, as early as after 3 months of NI, a significant increase of DD energy value associated with an increase of fat, protein and carbohydrates intakes was observed. The comparison of dancers’ diet before and after the 9-month NI revealed significant differences with regard to energy value and an increased intake of all macronutrients. Therefore, significant changes in EB and EA were observed. In addition, EA was over the critical level of 30 kcal//kg FFM/d. After three months of NI use in the group of dancers, no statistically significant changes in body composition were seen. After another 3-month period, differences in BW, BMI and FFM (kg) turned out to be significant, which was also maintained at 9-month time point. After 3 months of NI in the group of female athletes, apart from significant energy intake increase, an important increase of total protein and carbohydrates intake, eliminating discrepancies between TEE and energy intake, and simultaneously the EA increase over the critical value of 30 kcal/kg FFM/d were observed. In contrast to dancers, no significant changes in BW, BMI and body composition, during the nine-month NI in female athletes were seen (Table 2). Amenorrhea was diagnosed in 5 out of all eligible dancers, and oligomenorrhea in 16 subjects. The nine-month NI use resulted in regular menses recovery in 3 ballerinas and in the lower number of dancers with amenorrhea. At the study end, changes in hormone levels (increased LH) were visible. On the other hand, amenorrhea was diagnosed in 5 subjects from the group of female athletes, while oligomenorrhea was observed in 26 females. The nine-month NI resulted in regular menstruation recovery in 7 female athletes. As early as after 3 months of the NI, LH levels and LH to FSH ratio changes were visible (Table 3). As the 9-month NI did not result in resumption of regular menstruation in all examined subjects, EB, EA, BW and body composition values, as well as the levels of selected hormones, were compared between two groups created on the basis of the above mentioned criterion, i.e. recovery of regular menstrual cycle. Even before the NI start, statistically significant intergroup differences concerning EB, EA, FM and FFM values were observed. Additionally, women with potential recovery of the regular menstrual cycle had almost normal EB, higher EA, higher FM, and lower FFM. Discrepancies in the above mentioned parameters were also visible at the NI end, when higher rate of FM and no changes in BW were observed. Moreover, the nine-month NI use resulted in significant increase of LH levels in both groups, whereas in the group with restored menstruation this value was higher (Table 4).

**Table 1:** **Baseline characteristics of study participants M ± SD**

**Parameters**	**Ballet dancers**	**Female athletes**	**P-value**
**Baseline characteristics**			
Age (years)	17.1 ± 0.9	18.1 ± 2.6	NS
Age at menarche (years)	167.1 ± 4.5	13.0 ± 1.2	0.018
Age at the beginning of training (years)	6.7 ± 0.5	11.2 ± 3.5	< 0.001
Training period (years)	10.7 ± 1.2	6.8 ± 3.3	< 0.001
Number of training sessions per week (n/d)	5.2 ± 0.5	5.2 ± 1.0	NS
Hours of training per day (hours/d)	4.7 ± 1.1	4.0 ± 1.8	NS
Hours of training per week (hours/week)	24.9 ± 6.9	19.5 ± 7.2	0.009
RMR predicted (kcal/d)	1399 ± 60	1458 ± 56	< 0.001
RMR measured (kcal/d)	1198 ± 142	1354 ± 151	0.001
RMR measured/predicted × 100%	85.6 ± 9.1	92.8 ± 10.0	0.011
RMR measured – RMR predicted (kcal/d)	−201.0 ± 122.1	−105.0 ± 146.8	0.017
RMR/FFM (kcal/kg)	28.2 ± 3.6	29.0 ± 3.6	NS
**Hormonal parameters**			
TSH (μIU/mL)	1.96 ± 1.33	1.74 ± 0.80	NS
PRL (ng/mL)	16.38 ± 8.88	13.0 ± 9.33	NS
T (ng/dL)	72.95 ± 25.92	37.28 ± 21.85	< 0.001
SHBG (nmol/L)	71.62 ± 22.03	62.79 ± 41.91	NS
Leptin (ng/mL)	4.59 ± 2.78	6.25 ± 4.94	NS

**Table 2:** **Effects of the 9-month nutritional intervention on the energy and nutrients intake, TEE, BW and composition M ± SD**

**Ballet dancers**										
	**0**	**3**	**6**	**9**	**0 vs. 3**	**3 vs. 6**	**0 vs. 6**	**3 vs. 9**	**6 vs. 9**	**0 vs. 9**
Energy (kcal)	1640 ± 412	2130 ± 503	2300 ± 290	2368 ± 182	< 0.001	NS	< 0.001	0.03	NS	< 0.001
Fat (g)	57.0 ± 18.9	71.0 ± 19.7	72.6 ± 17.2	73.2 ± 15.9	<0.001	NS	<0.001	NS	NS	<0.001
Protein (g)	58.8 ± 13.3	77.3 ± 17.5	82.7 ± 9.7	85.3 ± 9.3	<0.001	NS	<0.001	NS	NS	<0.001
Carbohydrate (g)	222.8 ± 55.7	295.7 ± 72.0	328.9 ± 50.9	342.0 ± 33.2	<0.001	NS	<0.001	0.004	NS	<0.001
TEE (kcal/d)	2038 ± 279	2170 ± 372	2276 ± 236	2292 ± 245	NS	NS	0.005	NS	NS	0.002
EB (kcal/d)	−384 ± 267	−40 ± 267	24 ± 111	75 ± 147	NS	NS	<0.001	NS	NS	<0.001
EEE (kcal/d)	731 ± 207	758 ± 315	737 ± 129	750 ± 113	<0.001	NS	NS	NS	NS	NS
EA (kcal/kg FFM/d)	21.7 ± 7.2	32.8 ± 6.8	35.9 ± 5.5	37.2 ± 4.2	0.NS	NS	<0.001	0.017	NS	<0.001
BW (kg)	52.9 ± 5.8	53.2 ± 5.7	54.4 ± 5.0	54.2 ± 4.7	<0.001	0.001	<0.001	0.003	NS	0.001
BMI (kg/m^2^)	18.9 ± 1.7	19.0 ± 1.7	19.5 ± 1.5	19.4 ± 1.4	NS	0.001	<0.001	0.003	NS	0.001
FM (%)	18.7 ± 3.2	19.2 ± 3.2	19.5 ± 3.7	19.3 ± 3.3	NS	NS	NS	NS	NS	NS
FM (kg)	9.9 ± 2.2	10.2 ± 2.3	10.6 ± 2.4	10.5 ± 2.1	NS	NS	NS	NS	NS	NS
FFM (%)	81.3 ± 3.2	80.8 ± 3.2	80.5 ± 3.7	80.7 ± 3.3	NS	NS	NS	NS	NS	NS
FFM (kg)	43.0 ± 4.8	42.8 ± 4.6	43.8 ± 4.4	43.7 ± 4.0	NS	0.042	NS	0.040	NS	NS
**Female athletes**										
	**0**	**3**	**6**	**9**	**0 vs. 3**	**3 vs. 6**	**0 vs. 6**	**3 vs. 9**	**6 vs. 9**	**0 vs. 9**
Energy (kcal)	2354 ± 539	2588 ± 557	2803 ± 387	2800 ± 321	0.041	NS	< 0.001	NS	NS	< 0.001
Fat (g)	92.2 ± 27.5	84.2 ± 20.4	95.0 ± 19.7	87.4 ± 11.1	NS	0.034	NS	NS	NS	NS
Protein (g)	75.6 ± 14.8	85.5 ± 15.6	90.8 ± 12.7	93.0 ± 10.7	0.004	NS	<0.001	0.038	NS	<0.001
Carbohydrate (g)	305.4 ± 78.0	372.2 ± 86.3	395.9 ± 59.8	410.2 ± 56.9	<0.001	NS	<0.001	0.038	NS	<0.001
TEE (kcal/d)	2642 ± 348	2638 ± 421	2744 ± 348	2741 ± 328	NS	NS	NS	NS	NS	NS
EB (kcal/d)	−288 ± 477	−51 ± 224	59 ± 72	59 ± 89	0.002	NS	<0.001	NS	NS	<0.001
EEE (kcal/d)	959 ± 174	905 ± 337	898 ± 283	868 ± 275	NS	NS	NS	NS	NS	NS
EA (kcal/kg FFM/d)	52.9 ± 5.8	35.8 ± 12.3	41.0 ± 8.6	40.9 ± 8.2	0.011	0.001	<0.001	0.001	NS	<0.001
BW (kg)	59.3 ± 5.3	59.6 ± 5.3	59.5 ± 5.7	59.4 ± 5.8	NS	NS	NS	NS	NS	NS
BMI (kg/m^2^)	20.6 ± 1.4	20.7 ± 1.5	20.7 ± 1.6	20.6 ± 1.7	NS	NS	NS	NS	NS	NS
FM (%)	20.6 ± 3.7	21.0 ± 3.5	21.3 ± 3.4	21.2 ± 3.1	NS	NS	NS	NS	NS	NS
FM (kg)	12.2 ± 2.4	12.5 ± 2.4	12.7 ± 2.7	12.6 ± 2.5	NS	NS	NS	NS	NS	NS
FFM (%)	79.4 ± 3.7	79.0 ± 3.7	78.7 ± 3.4	78.8 ± 3.1	NS	NS	NS	NS	NS	NS
FFM (kg)	47.1 ± 4.9	47.1 ± 4.8	46.8 ± 4.2	46.8 ± 4.3	NS	NS	NS	NS	NS	NS

0 - before nutritional intervention, 3 – after 3 months of the nutritional intervention, 6 – after 6 month of the nutritional intervention, 9 – after 9 month of the nutritional intervention (post –study).

**Table 3:** **Hormone levels in study participants after and during the nutritional intervention M ± SD**

	**0**	**3**	**6**	**9**	***P-value***					
					**0 vs 3**	**3 vs 6**	**0 vs 6**	**3 vs 9**	**6 vs 9**	**0 vs 9**
**Ballet dancers**										
Eumenorrhea	0	0	1	3						
Amenorrhea	5	4	3	1						
Oligomenorrhea	16	17	17	17						
LH (mlU/mL)	3.00 ± 1.51	4.17 ± 2.29	4.63 ± 2.47	4.81 ± 1.64	NS	NS	NS	NS	NS	0.026
FSH (mlU/mL)	5.23 ± 2.32	4.95 ± 1.67	4.67 ± 2.16	4.90 ± 1.08	NS	NS	NS	NS	NS	NS
E_2_ (pg/mL)	39.9 ± 21.7	43.7 ± 23.6	42.0 ± 20.0	42.3 ± 18.9	NS	NS	NS	NS	NS	NS
P (ng/mL)	0.53 ± 0.50	0.41 ± 0.63	0.24 ± 0.12	0.25 ± 0.09	NS	NS	NS	NS	NS	NS
LH to FSH ratio	0.72 ± 0.54	0.81 ± 0.49	1.03 ± 0.57	0.92 ± 0.22	NS	NS	NS	NS	NS	NS
**Female athletes**										
Eumenorrhea	0	0	4	7						
Amenorrhea	5	5	5	4						
Oligomenorrhea	26	26	22	20						
LH (mlU/mL)	3.04 ± 1.63	4.59 ± 2.53	4.67 ± 2.49	4.89 ± 1.99	0.009	NS	0.005	NS	NS	0.001
FSH (mlU/mL)	5.01 ± 2.37	5.00 ± 2.08	4.99 ± 2.01	5.04 ± 1.86	NS	NS	NS	NS	NS	NS
E_2_ (pg/mL)	36.5 ± 19.4	36.2 ± 15.3	39.2 ± 18.4	34.4 ± 11.7	NS	NS	NS	NS	NS	NS
P (ng/mL)	0.54 ± 0.99	0.68 ± 0.77	0.46 ± 0.32	0.36 ± 0.38	NS	NS	NS	NS	NS	NS
LH to FSH ratio	0.84 ± 0.56	0.96 ± 0.52	0.98 ± 0.49	0.97 ± 0.29	0.001	NS	0.003	NS	NS	0.033

0 - before nutritional intervention, 3 – after three month of the nutritional intervention, 6 – after six month of the nutritional intervention 9 – after nine month of the nutritional intervention (post – study).

**Table 4:** **Baseline and post-study characteristic among study participants with and without return of menses M ± SD**

	**Return of menses (n = 10)**			**No return of menses (n = 42)**			**0 vs. 0**	**9 vs. 9**
	**0**	**9**	**0 vs. 9**	**0**	**9**	**0 vs. 9**	**p**	**p**
			**p**			**p**		
EB (kcal/day)	−44 ± 508	121 ± 188.0	NS	−395 ± 352.7	52 ± 87.5	< 0.001	0.01	NS
EA (kcal/kg FFM/d)	31.0 ± 8.3	42.0 ± 7.8	<0.001	24.5 ± 8.8	38.9 ± 6.9	<0.001	0.05	NS
BW (kg)	57.4 ± 4.8	57.5 ± 4.1	NS	56.6 ± 6.6	57.2 ± 6.8	NS	NS	NS
BMI (kg/m^2^)	20.7 ± 1.2	20.8 ± 0.9	NS	19.7 ± 1.9	19.9 ± 2.0	NS	NS	NS
FM (%)	22.0 ± 2.9	22.8 ± 0.9	NS	19.3 ± 3.6	19.9 ± 3.4	NS	0.04	0.01
FM (kg)	12.6 ± 2.0	13.1 ± 1.1	NS	10.9 ± 2.6	11.4 ± 2.7	NS	NS	NS
FFM (%)	78.0 ± 2.9	77.2 ± 0.9	NS	81.7 ± 3.6	80.1 ± 3.7	NS	0.04	0.01
FFM (kg)	44.8 ± 4.0	44.4 ± 3.2	NS	45.7 ± 5.5	45.8 ± 4.7	NS	NS	NS
LH (mlU/mL)	2.78 ± 1.60	5.33 ± 1.97	0.016	3.22 ± 1.71	4.53 ± 1.61	0.002	NS	NS
FSH (mlU/mL)	5.02 ± 2.38	6.08 ± 1.91	NS	5.16 ± 2.34	5.19 ± 1.53	NS	NS	NS

0 - before nutritional intervention, 9 – after 9 month of the nutritional intervention (post – study).

## Discussion

Our results confirm the role of a non-pharmacological NI comprising increased energy and nutrition intakes and dietitian consultations, without any TEE changes, in treatment not only of female athletes, but also dancers with hypothalamic amenorrhea and oligomenorrhea.

The evaluation of subject’s eating habits performed prior to the beginning of the NI, indicated deficiencies in energy and nutritional values of DD which may, among others, impair the endocrine functions of the FM associated with leptin secretion, and therefore lead to menstrual disturbances [17]. In 90.5% of ballet dancers and 64.5% of female athletes the serum leptin levels were below the laboratory norms what is consistent with results obtained by other authors [18,19]. However, in 9.5% of ballet dancers and 29% of female athletes higher leptin levels were observed. It has been reported that a diet composition has a significant effect on the leptin synthesis, whereas in subjects on a high-energy diet higher leptin levels may be observed. Therefore, it may explain higher leptin levels in some female athletes because this group is known to use the type of a diet mentioned above. Mantzoros et al. [20] report that late high-energy evening meal consumption may also induce an increase in the leptin levels by 40-60%, persisting up to 12 hours. Despite the fact that all subjects were instructed not to consume high-energy meals late in the evening on the day before blood collection, it was impossible to verify their compliance. Genetic factors may also have a significant impact on the leptin levels. Therefore, it is difficult to unanimously determine the cause of high leptin levels observed in some subjects. Moreover, not all factors responsible for the serum leptin levels are known.

Energy intake restrictions are also regarded as the main reason for RMR reduction [21,22]. This study also demonstrates significant intergroup differences in the RMR rate. In ballet dancers, compared to female athletes, EB and EA were lower what potentially confirms the diet impact on the RMR value. The above-mentioned results are similar to those obtained by Lebenstedt et al. [23] who in female athletes with menstrual disturbances demonstrated lower RMR values by 114 kcal/d. Also Myburgh et al. [24] reported that reduced RMR, observed in dancers, was associated with menstrual disorders. Kaufman et al. [25] demonstrated that the RMR depended not only on diet composition, but also on the leptin levels. Moreover, Welt et al. [26] claimed that the insufficient energy intake and low serum leptin levels resulted in synthesis and frequency of gonadotropic hormone pulses reduction, and thus lead to ovulation inhibition. Furthermore, Kelesidis et al. [27] stated that the leptin deficit was responsible for reduced LH secretion and delayed menarche in patients with menstruation disorders. A similar conclusion was emphasized by Ackerman et al. [18] who indicated that low leptin levels may contribute to changes in the LH pulse frequency resulting in amenorrhoea.

During a nutritional intervention, an individual meal schedule was based on the real energy expenditure, current nutritional status, and in case of female athletes, on a current training stage. After a NI had started, the intervention was followed up every month. This follow-up demonstrated significant changes in a subject’s diet (a statistical analysis comprised data obtained before the start and 3, 6 and 9 months after the intervention start). Mean values indicated an energy and nutrient intake increase and high standard deviations; they also indicated that not all dancers and female athletes followed NI guidelines what indicates an important difficulty associated with implementation of an individual diet in this population. However, in subsequent NI follow-ups, the standard deviation value was gradually lower indicating progressive implementation of dietetic changes. However, the rate of such changes was different than expected. Thus, in both dancers and female athletes the EB value was compensated and the EA level exceeded the critical threshold of 30 kcal/kg FFM/d, below which, according to other authors [28,29], the menstruation disorders become more intense and the risk of bone mineral density reduction increases. Unfortunately, even after 9 months of the NI use the recommended level of vitamin D intake was not achieved neither in dancers nor in female athletes. This could be explained by a high demand standard accepted based on the ACSM recommendations [6,30,31]. However, the principal aim of this paper was to follow the guidelines of an individual diet without the need for additional vitamin and mineral supplementation. Nevertheless, in dancers and female athletes at the risk of a triad syndrome, additional vitamin D supplementation may be necessary in the nearest future.

In our study, the return of menses was observed in 3 ballet dancers and 7 female athletes. Moreover, after a 9-month NI there were less subjects with secondary amenorrhea. Among hormones responsible for the maintenance of a regular menstrual cycle a significant increase in the LH levels was observed in dancers on the third follow-up visit monitoring the effects of a nutritional intervention (after 9 months of the NI). On the other hand, in female athletes beneficial changes in the levels of this hormone were observed as early as after the third month of a diet. Results obtained may indicate that increased energy and nutrient consumption has a positive effect on the hormone status in young women with menstruation disorders. It is extremely important to note that a 9-month diet used in the group of female athletes did not result in any changes of the BW value and body composition. However, in a group of ballet dancers an increase in the BW and BMI values was visible as early as after a 3-month diet and resulted in an FFM increase. Changes of the body weight in a group of ballet dancers during a 9-month nutritional intervention were 1.3 kg. It has to be emphasised that a more negative energy balance and lower energy availability were observed in this group before the start of the nutritional intervention. Additionally, ballet dancers had a lower resting metabolism rate. Results obtained may indicate that this group is malnourished to a greater extent and it may account for more significant changes in their body weight. The comparison of a nutritional status, EB and EA values between a group with recovered menses and a group with menstrual disturbances suggests that resumption of menstruation can be obtained when the FM level reaches a specific value, i.e. 22%. This observation seems to confirm the hypothesis of Frish et al. [32] who showed that a BW increase alone was not so important. These findings are inconsistent with prior studies conducted by Zanker et al. [33] who demonstrated an association between BW and resumption of menses in female athletes with hypothalamic amenorrhea. In their study, Arends et al. [3] demonstrated that regular menstruation recovery in female athletes depends on the BW and BMI increase. Unfortunately, the publication by Arends et al. [3] does not provide the parameters of female athletes’ body composition, therefore a full comparison of those results with results obtained herein is impossible. It is difficult to overestimate practical implications of our observations confirming that a relatively low difference in the FM amount, potentially obtained without a significant BW increase, may result in regular menstruation recovery. Our studies included girls practicing disciplines in which a silhouette is of vital importance. Thus, the NI implementation in dancers was a significant problem due to a belief that a BW increase may potentially translate into subjective ratings received from judges and lower scores for visual performance and artistic image; while in triathlonists or rowers it may reduce their speed or physical capability. In our study, a 9-month nutritional intervention, similarly to other studies, was used. Dueck et al. [34] and Knopp-Woodroffe et al. [35] demonstrated menses resumption in female athletes after approximately 6 months and 9–12 weeks, respectively. However, based on our results, we can suggest that the mean time to menses recovery could be longer than 1 year. Also Arends et al. [3] suggested that a non-pharmacological intervention in female athletes with menstrual disturbances might restore regular cycles, although the restoration of menses may take more than 1 year.

It may be suggested that limitations associated with regular menses restoration in the study population, both in our paper and in other authors’ studies, may be due to the fact that there are many overlapping factors contributing to above-mentioned disturbances. Bruni et al. [36] state that an inappropriate diet, increased physical effort and stress are basic factors differentiating women suffering from menstruation disturbances. Therefore, it is not recommended to combine the general population suffering from such conditions into one group. In our studies, young eligible women form a specific population in which all factors predisposing to above-mentioned dysfunctions seem to be combined. Papers by Nattiv et al. [6] and Manore et al. [37] emphasized that an appropriately balanced diet with simultaneous limitation of the training volume and intensity is one of many potential possibilities that may be used to reduce menstrual disorders in dancers and female athletes. The above-mentioned papers were significant because they emphasized an appropriately planned and individualized diet including daily load with physical training. Moreover, the effects were obtained without any changes in the volume and intensity of training, taking into account the fact that it might have been an argument against participation in these studies.

## Conclusion

Menstruation disorders reduction, in dancers and female athletes, is possible without any pharmacological intervention only by following the guidelines of an appropriately planned NI comprising balanced energy balance and higher energy availability. However, time period duration with possibility of complete menstruation recovery, due to non-pharmacological NI use, depends to a large extent on women’s “baseline characteristics” including their body composition, nutritional status, earlier nutritional habits and metabolic state. When the baseline amount of FM is low, i.e. below 20%, the need of longer NI use, even >1 year, should be considered. The regulation of gonadotropic hormone levels alone may not translate into immediate restoration of regular menses, although it would definitely make menstrual disorders less severe. Further research is needed in larger samples to determine the primary contributors to resumption of menses in amenorrheic and oligomenorreic, exercising women.
